# Computed tomography-based radiomics improves non-invasive diagnosis of *Pneumocystis jirovecii* pneumonia in non-HIV patients: a retrospective study

**DOI:** 10.1186/s12890-023-02827-4

**Published:** 2024-01-02

**Authors:** Hang Yu, Zhen Yang, Yuanhui Wei, Wenjia Shi, Minghui Zhu, Lu Liu, Miaoyu Wang, Yueming Wang, Qiang Zhu, Zhixin Liang, Wei Zhao, Liang-an Chen

**Affiliations:** 1 Department of Respiratory and Critical Care Medicine, Medical School of Chinese People’s Liberation Army, Beijing, China; 2 Department of Respiratory and Critical Care Medicine, the Eighth Medical Center, Chinese People’s Liberation Army General Hospital, Beijing, China; 3 Department of Pulmonary and Critical Care Medicine, Zhongnan Hospital of Wuhan University, Wuhan, Hubei China; 4 Department of Nutrition, the First Medical Center, Chinese People’s Liberation Army General Hospital, Beijing, China

**Keywords:** *Pneumocystis Jirovecii* Pneumonia, Computed tomography, Radiomics, Diagnostic tests

## Abstract

**Background:**

*Pneumocystis jirovecii* pneumonia (PCP) could be fatal to patients without human immunodeficiency virus (HIV) infection. Current diagnostic methods are either invasive or inaccurate. We aimed to establish an accurate and non-invasive radiomics-based way to identify the risk of PCP infection in non-HIV patients with computed tomography (CT) manifestation of pneumonia.

**Methods:**

This is a retrospective study including non-HIV patients hospitalized for suspected PCP from January 2010 to December 2022 in one hospital. The patients were randomized in a 7:3 ratio into training and validation cohorts. Computed tomography (CT)-based radiomics features were extracted automatically and used to construct a radiomics model. A diagnostic model with traditional clinical and CT features was also built. The area under the curve (AUC) were calculated and used to evaluate the diagnostic performance of the models. The combination of the radiomics features and serum β-D-glucan levels was also evaluated for PCP diagnosis.

**Results:**

A total of 140 patients (PCP: *N* = 61, non-PCP: *N* = 79) were randomized into training (*N* = 97) and validation (*N* = 43) cohorts. The radiomics model consisting of nine radiomic features performed significantly better (AUC = 0.954; 95% CI: 0.898-1.000) than the traditional model consisting of serum β-D-glucan levels (AUC = 0.752; 95% CI: 0.597–0.908) in identifying PCP (*P* = 0.002). The combination of radiomics features and serum β-D-glucan levels showed an accuracy of 95.8% for identifying PCP infection (positive predictive value: 95.7%, negative predictive value: 95.8%).

**Conclusions:**

Radiomics showed good diagnostic performance in differentiating PCP from other types of pneumonia in non-HIV patients. A combined diagnostic method including radiomics and serum β-D-glucan has the potential to provide an accurate and non-invasive way to identify the risk of PCP infection in non-HIV patients with CT manifestation of pneumonia.

**Trial registration:**

ClinicalTrials.gov (NCT05701631).

**Supplementary Information:**

The online version contains supplementary material available at 10.1186/s12890-023-02827-4.

## Background

*Pneumocystis jirovecii* pneumonia (PCP) is an opportunistic lung infection caused by *P. Jirovecii* that usually affects immunocompromised patients with or without human immunodeficiency virus (HIV) infection [1, 2]. In recent years, the incidence of PCP has been increasing in non-HIV, with a significantly higher mortality rate (17.2-52.9%) than in HIV patients (mortality rate: 6.7%) [3, 4]. PCP is initially suspected on the basis of symptoms (fever, cough, dyspnea), computed tomography (CT) findings (e.g., ground glass opacities) and high risk factors (an underlying immunodeficiency) [5, 6]. The diagnosis is confirmed by identification of cysts or trophozoites from bronchoalveolar lavage (BAL) or biopsy by direct immunofluorescence (IF) or conventional staining [7]. Real-time quantitative polymerase chain reaction (qPCR) testing on specimens is another method recommended by guidelines but should be used in combination with IF staining to improve its specificity [8]. Unfortunately, diagnosis is challenging, as bronchoscopy or biopsy can lead to complications such as fever, worsening hypoxemia or the need for tracheal intubation and mechanical ventilation [9, 10]. Meanwhile, these diagnostic methods are time-consuming and cannot provide early indication of PCP infection risk. Non-invasive methods, such as qPCR and/or IF staining on induced sputum, oral washings, nasopharyngeal aspirate are not recommended due to unsatisfactory diagnostic accuracy [8, 11]. Serum β-D-glucan detection is also considered as a supplementary means as it requires a high pretest probability [8, 11, 12]. Therefore, there is an urgent need to explore new technologies that can detect the risk of PCP infection early, accurately, and non-invasively to guide clinical interventions.

Radiomics is a new method of image processing that has emerged in the last decade. By transforming images into massive amounts of data, extensive features invisible to the naked eye can be extracted and analysed for diagnosis, severity assessment and prognosis of diseases [13, 14]. In recent years, the application of radiomics has gradually expanded from oncology research to others [15, 16]. In particular, radiomics models based on computed tomography (CT) have demonstrated great performance in the diagnosis and prognosis of COVID-19 pneumonia [17, 18]. However, there are few studies focusing on the radiomic features of PCP, especially in non-HIV patients [19]. In the present study, we aim to investigate the radiomics features of PCP and develop a radiomics-based model to provide an early indication of the risk of PCP infection in non-HIV patients.

## Methods

### Study population

**Fig. 1:**
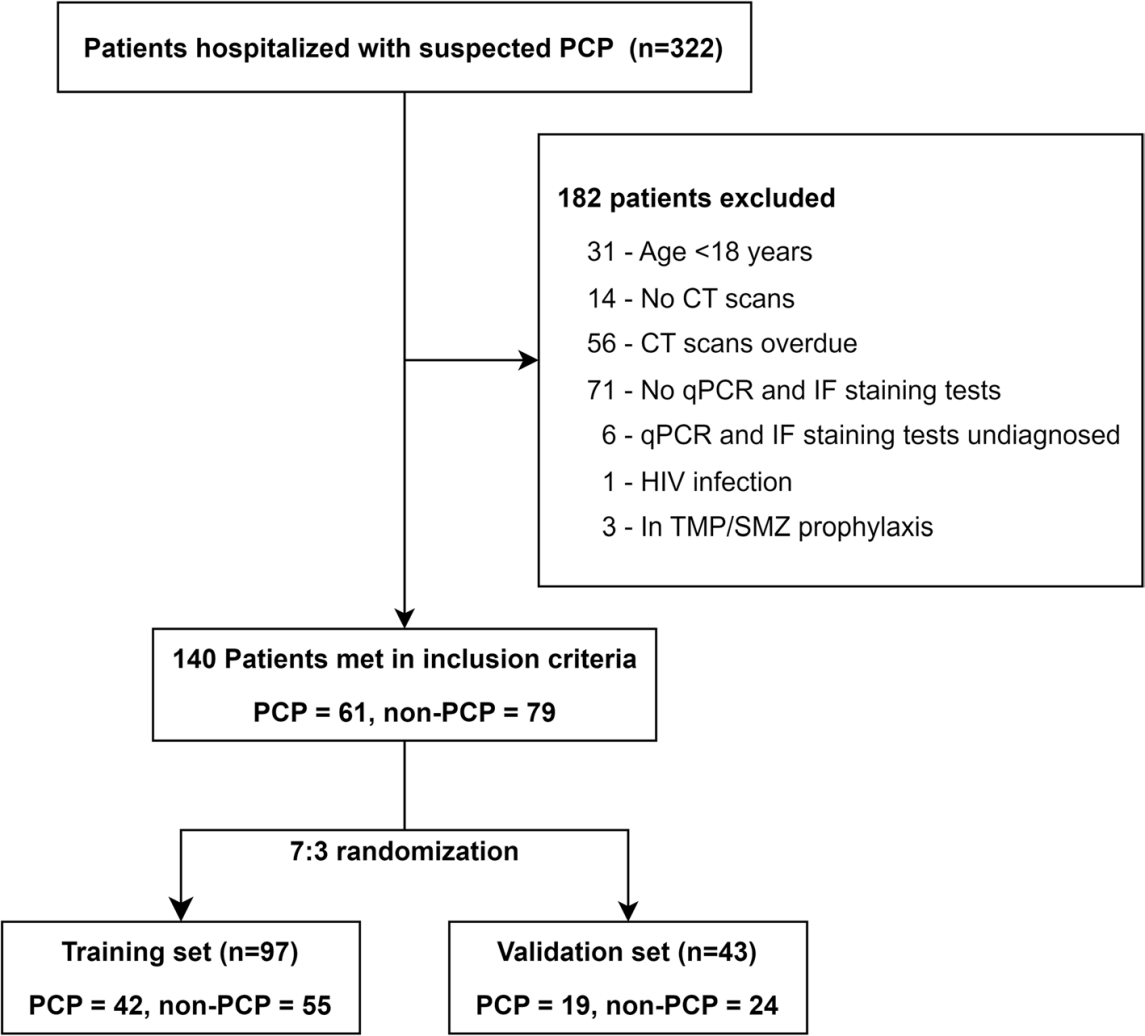
Flow chart of the study. PCP, *Pneumocystis jirovecii* pneumonia; CT, computed tomography; qPCR, quantitative polymerase chain reaction; IF, immunofluorescent; HIV, human immunodeficiency virus; TMP/SMZ, trimethoprim-sulfamethoxazole

This study was approved by the Ethics Committee of Chinese PLA General Hospital (NO. S2023-006-01) and was conducted in accordance with the Declaration of Helsinki (as revised in 2013). The study was registered in ClinialTrials.gov (27/01/2023, NCT05701631). Individual informed consent for this retrospective analysis was waived by the Ethics Committee of Chinese PLA General Hospital. From January 2010 to December 2022, patients admitted to our institute with clinical suspected PCP were screened for inclusion. The inclusion criteria were as follows: (I) aged over eighteen years; (II) presence of underlying immunodeficiency reported to be associated with PCP, including autoimmune diseases, hematological malignancies, solid cancers, transplantation, corticosteroid use, immunosuppressants use and chemotherapeutic agents use [5, 20]; (III) symptoms of lower respiratory tract infection, such as fever, cough or dyspnea; (IV) signs of lung infection on high resolution CT at the on-set of the disease, including ground glass opacity, consolidation, honeycombing, interlobular septal thickening and pleural effusion [14]; (V) received BAL examination within three days after CT scans; (VI) underwent qPCR and IF staining tests on the BAL fluid sample. Patients with HIV infection, those taking trimethoprim-sulfamethoxazole for prophylaxis, or those undiagnosed by qPCR and IF staining tests were excluded. In total, 322 patients were evaluated, and 140 patients were included in this study who were then randomized at a 7:3 radio into training (*N* = 97) and a validation set (*N* = 43) (Fig. 1).

### Clinico-demographic data collection

The clinical, laboratory and CT image data were retrospectively gathered through our institute’s medical record system. The collected clinical features included age, sex, smoking history, underlying diseases, medication use including corticosteroid, immunosuppressant or chemotherapeutic agents use, clinical symptoms including fever, cough, dyspnea; laboratory findings including PaO_2_/FiO_2_ ratio measured on arterial blood, white blood cell, serum c-reaction protein, serum lactate dehydrogenase (LDH), and serum β-D-glucan.

### CT scanning protocols

Chest CT scans were carried out in a CT scanner (SOMATOM Definition AS+, Siemens Healthcare, Forchheim, Germany). Scanning parameters were as follows: tube voltage of 120 kV, automatic exposure control, tube rotational speed of 0.5 s/rot, collimation of 0.6 × 64 mm, pitch of 0.984, matrix size of 512 × 512 mm, reconstructed slice thickness of 1-1.25 mm and reconstructed kernel of B70f. All the images used for analysis were unenhanced.

### Segmentation and radiomic features extraction

**Fig. 2:**
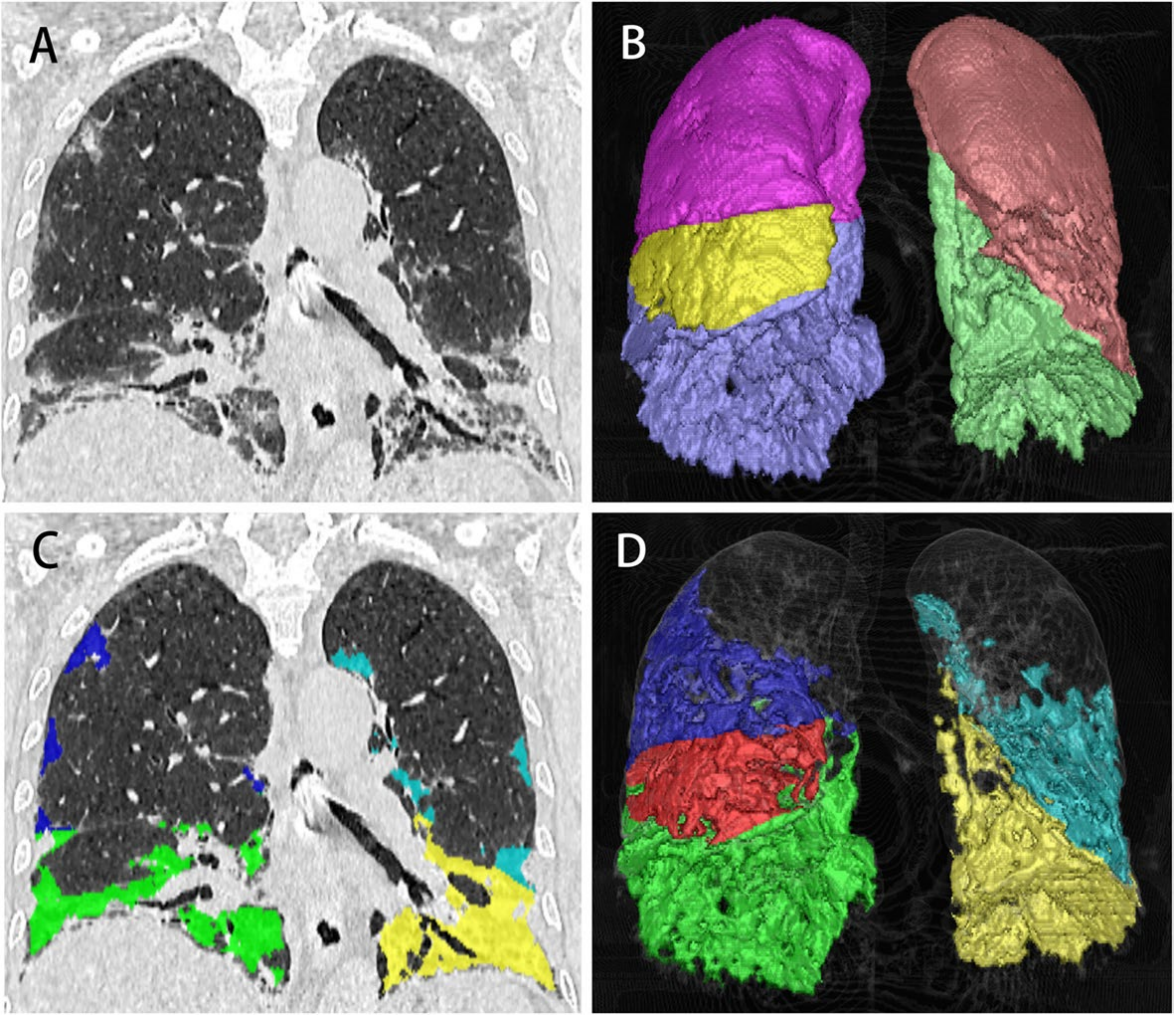
The segmentation of infected areas (i.e., the region of interest, ROI) on the FACT Medical Imaging System. A 59-year-old female with anti-synthetase syndrome and a long history of corticosteroid use was admitted to hospital with fever and cough for 7 days. **A** Sagittal HRCT images showed ground-glass opacity and thickening interlobular septal predominantly in both lower lungs. **B** Three-dimensional (3D) volume rendering image of the lobes (purple: right upper lobe; yellow: right middle lobe; blue: right lower lobe, brown: left upper lobe; green: left lower lobe). **C** and **D** Sagittal CT and 3D images of automatically identified infected areas. Different colors indicated that the infection was in different lobes (blue: right upper lobe; red: right middle lobe; green: right lower lobe; cyan: left upper lobe; yellow: left lower lobe). The ROIs are integrated as one and radiomic features were extracted from it

Image segmentation was performed by -a Food and Drug Administration (FDA) approved imaging software of FACT medical imaging system (Version 1.5, Dexin Medical Imaging Technology Company). Firstly, the pneumonia regions of the CT data were identified and segmented automatically by using a previously reported deep learning algorithm [21, 22]. The average density of the lung parenchyma was used to compute a threshold (the lowest density) to detect CT abnormalities including ground glass opacity, consolidation, honeycombing and interlobular septal thickening. The detected abnormalities of the whole lung were masked as the region of interest (ROI) in the automatic processing mode of “Pneumonia” (Fig. 2). Then, an independent senior respiratory physician (WZ with fifteen years of experience in lung CT imaging) reviewed the segmentation results in a fixed lung window (level: -500HU; width: 1500HU) and made modifications if necessary. An expert in chest radiology (WY) confirmed the results. Then, the region of interest was resampled at 1 mm × 1 mm × 1 mm and 1316 radiomic features were extracted using the “Radiomics” module and normalized through Z-Score method. The features included: (I) shape; (II) first order features, (III) texture features, (IV) features extracted from filtered images, i.e., wavelet and Laplacian of Gaussian (LoG) features. In total, 37 ROIs of 12 patients (9%) were manually modified, including 13 unidentified and 24 inaccurate pneumonia regions. The modification time for one patient was approximately 30 to 65 min. For patients whose ROIs need no manual modification, each CT DICOM file was processed in 6–10 min.

### Radiomics model construction

First, Student’s t-test and Mann-Whitney U-test were used to identify the significant features in the training cohort. The Least Absolute Shrinkage and Selection Operator (LASSO) were then used and features with non-zero coefficients were identified at the optimal regularization parameter (λ) by tenfold cross-validation. After that, a radiomics model were constructed using logistic regression. The area under the curve (AUC) of the receiver operating characteristic (ROC) curves were calculated and used to evaluate the diagnostic performance of the model. In addition, the radiomic score (Radscore) was calculated, and calibration curves and a waterfall plot of the Radscore were plotted to show the diagnostic accuracy of the model.

### Traditional risk factors model construction

The CT images were viewed and evaluated at a fixed lung window (level: -500 HU; width: 1500 HU) by two independent respiratory physicians (QZ, ZL) with eight and twenty years of experience in lung CT image reading. An expert chest radiologist (WY) confirmed the results. The CT abnormalities included ground-glass opacity, interlobular septal thickening, cyst, consolidation, honeycombing, mediastinal lymphadenopathy, and pleural effusion. All CT signs are identified according to the definitions in the glossary of terms published by the Fleischner Society in 2008 [23]. Semantic CT features and clinical characteristics significantly associated with the diagnosis of PCP were screened by univariate analysis and used for multivariate logistic regression analysis. The independent factors identified by multivariate logistic regression were then used to construct a traditional model. The AUC was calculated in both training and validation cohorts and compared with the radiomics model by the Delong test.

### Clinical utility of the models

The clinical utility of the radiomics model and the traditional model were assessed with decision curve analysis by calculating the net benefit at different threshold probabilities.

### Diagnostic performance comparison with serum β-D-glucan and LDH

Previous-reported risk factors for PCP infection, including serum β-D-glucan > 200 pg/mL or LDH > 300 IU/mL as PCP positive, and β-D-glucan < 80 pg/mL as PCP negative, were also evaluated for diagnostic performance among all patients [1, 12, 24, 25]. Accuracy, sensitivity, specificity, positive predictive value (PPV) and negative predictive value (NPV) were calculated and compared with the radiomics model.

### Standard of reference

Real-time qPCR and IF staining tests were performed on BAL fluid samples to detect *P. Jirovecii.* QPCR > 0 pathogens/mL was considered as qPCR (+). IF staining was considered positive when *P. Jirovecii* cysts or trophozoites were found. PCP diagnosis was made based on the results of qPCR and IF staining tests according to the criteria developed by the European Conference on Infection in Leukaemia (ECIL) guidelines 2016 [8].

### Statistical analysis

The R software (version 4.2.2, The Free Software Foundation, USA) and the SPSS for Windows, version 26.0 (IBM Corp., Armonk, N.Y., USA) were used for randomization, model construction and statistical analysis. The “glmnet”, “e1071”, and “adabag” packages were used for model construction by LASSO regression, support vector machine and adaboost. The “pROC”, “ggplot2”and “rmda” packages were used to plot ROC and decision curves. The “rms”, “Hmisc”, “Survival”, “Lattice”, “Formula” and “waterfalls” package were used to draw calibration curve and waterfall plots. Depending on the type and distribution of the data, Student’s t-test, Mann-Whitney U-test or Pearson’s x^2^ test were applied to test for the significance of between-group differences in clinical characteristics and semantic CT features. The Delong test was used to compare the differences between AUCs of ROC curves of different models. *P*-values less than 0.05 were considered statistically significant.

## Results

### Patient characteristics

A total of 140 patients were included in this study (Fig. 1; Table 1). Sixty-one patients were diagnosed with PCP (as the PCP group) and 79 with other types of pneumonia (as the non-PCP group). The other types of pneumonia included: bacterial pneumonia (*n* = 43), viral pneumonia (*n* = 14), fungal pneumonia (*n* = 5), radiation pneumonia (*n* = 2), and interstitial pneumonia associated with connective tissue disease (*n* = 15). In the PCP group, the proportion of ex-smokers was higher (36.1%, *P* = 0.038), and more patients used corticosteroids (73.8%, *P* < 0.001) or immunosuppressants (54.1%, *P* < 0.001. Meanwhile, the PCP patients had significant lower PaO_2_/FiO_2_ ratio (*P* = 0.004) but higher serum C-reaction protein (*P* = 0.004), LDH (*P* < 0.001) and β-D-glucan (*P* < 0.001). For semantic CT features, there are more ground glass opacity (100%, *P* = 0.002) and cyst (31.1%, *P* = 0.041) in the PCP group. In the non-PCP group, more patients used chemotherapeutic agents (46.8%, *P* = 0.004), and the proportion of patients with a CT sign of pleural effusion was higher (27.8%, *P* = 0.038).

**Table 1:** Comparison of clinical characteristics and semantic CT features in *Pneumocystis jirovecii* pneumonia (PCP) and other types of pneumonia (non-PCP) group

Characteristics	Total	PCP group	non-PCP group	*P* value
	(*N* = 140)	(*N* = 61)	(*N* = 79)	
Age, years, median (IQR)	51 (32, 58)	53 (38, 58)	47 (30, 58)	0.119
Sex, female, n (%)	59 (42.1%)	22 (36.1%)	37 (46.8%)	0.202
Ever-smokers, n (%)	38 (27.1%)	22 (36.1%)	16 (20.3%)	0.038*
Underlying diseases, n (%)				
Autoimmune diseases	51 (36.4%)	20 (32.8%)	31 (39.2%)	NA
Hematological malignancies	46 (32.9%)	10 (16.4%)	36 (45.6%)	NA
Solid cancers	4 (2.9%)	3 (4.9%)	1 (1.3%)	NA
Transplantation	15 (10.7%)	11 (18.0%)	4 (5.1%)	NA
Renal diseases	14 (10.0%)	12 (19.7%)	2 (2.5%)	NA
Else	10 (7.1%)	5 (8.2%)	5 (6.3%)	NA
Corticosteroid use, n (%)	71 (50.7%)	45 (73.8%)	26 (32.9%)	< 0.001***
Immunosuppressants use, n (%)	52 (37.1%)	33 (54.1%)	19 (24.1%)	< 0.001***
Chemotherapeutic agents use, n (%)	51 (36.4%)	14 (23.0%)	37 (46.8%)	0.004**
Initial symptoms, n (%)				
Fever	87 (62.1%)	43 (70.5%)	44 (55.7%)	0.075
Cough	63 (45.0%)	28 (45.9%)	35 (44.3%)	0.851
Dyspnea	69 (49.3%)	39 (63.9%)	30 (38.0%)	0.002**
Initial laboratory findings, median (IQR)				
PaO_2_/FiO_2_ ratio	279 (259, 298)	249 (219, 279)	305 (281, 330)	0.004**
WBC, 10^9/L	6.74 (4.75, 9.44)	7.04 (5.06, 8.98)	6.47 (4.22, 10.38)	0.530
CRP, mg/L	1.52 (0.29, 5.67)	2.28 (0.77, 7.02)	1.21 (0.17, 3.08)	0.004**
serum LDH, IU/L	313 (226, 429)	367 (269, 527)	262 (181, 354)	< 0.001***
serum β-D-glucan, pg/mL	50.1 (13.3, 290.3)	273.4 (102.6, 512.3)	20.6 (10.0, 50.2)	< 0.001***
Semantic CT features, n (%)				
Ground glass opacity	129 (92.1%)	61 (100.0%)	68 (86.1%)	0.002**
Interlobular septal thickening	58 (41.4%)	28 (45.9%)	30 (38.0%)	0.347
Cyst	32 (22.9%)	19 (31.1%)	13 (16.5%)	0.041*
Consolidation	67 (47.9%)	25 (41.0%)	42 (53.2%)	0.154
Honeycombing	16 (11.4%)	9 (14.8%)	7 (8.9%)	0.279
Lymphadenopathy	28 (20.0%)	11 (18.0%)	17 (21.5%)	0.610
Pleural effusion	30 (21.4%)	8 (13.1%)	22 (27.8%)	0.036*

*Abbreviations:* *IQR* Interquartile range, *PaO*_*2*_ Partial pressure of oxygen, *FiO*_*2*_ Fraction of Inspired oxygen, *WBC* White blood cell, *CRP* C-reaction protein, *LDH* Lactate dehydrogenase

*P<0.05; **P<0.01; ***P<0.001

All patients were randomized into a training cohort (*N* = 97) and a validation cohort (*N* = 43) (Table 2). In the training cohort, significant differences were found between the PCP and non-PCP group in terms of medication use, laboratory findings (c-reaction protein, LDH, β-D-glucan) and CT features (ground glass opacity). Multivariate logistic regression revealed that the serum β-D-glucan was the only independent factor associated with the diagnosis of PCP (Odds ratio: 1.010; 95% CI: 1.006–1.015; *P* < 0.001). The AUCs of the serum β-D-glucan (as a traditional model) were 0.859 (95% CI: 0.774–0.944) and 0.752 (95% CI: 0.597–0.908) in the training and validation cohorts, respectively.

**Table 2:** Comparison of clinical characteristics and semantic CT features in patients in the training and validation set

Characteristics	Training set (*n* = 97)		*P* value	Validation set (*n* = 43)		*P* value
	PCP	non-PCP		PCP	non-PCP	
	(*N* = 42)	(*N* = 55)		(*N* = 19)	(*N* = 24)	
Age, years, median (IQR)	53 (45, 59)	48 (32, 57)	0.092	51 (30, 58)	43 (24, 59)	0.741
Sex, female, n (%)	17 (40.5%)	23 (41.8%)	0.895	5 (26.3%)	14 (58.3%)	0.038*
Ever-smokers, n (%)	16 (38.1%)	13 (23.6%)	0.125	6 (31.6%)	3 (12.5%)	0.131
Underlying diseases, n (%)						
Autoimmune diseases	15 (35.7%)	22 (40.0%)	NA	5 (26.3%)	9 (37.5%)	NA
Hematological malignancies	6 (14.3%)	26 (47.3%)	NA	4 (21.1%)	10 (41.7%)	NA
Solid cancers	2 (4.8%)	1 (1.8%)	NA	1 (5.3%)	0 (0%)	NA
Transplantation	5 (11.9%)	3 (5.5%)	NA	6 (31.6%)	1 (4.2%)	NA
Renal diseases	9 (21.4%)	1 (1.8%)	NA	3 (15.8%)	1 (4.2%)	NA
Else	5 (11.9%)	2 (3.6%)	NA	0 (0%)	3 (12.5%)	NA
Corticosteroid use, n (%)	33 (78.6%)	17 (30.9%)	< 0.001***	12 (63.2%)	9 (37.5%)	0.099
Immunosuppressants use, n (%)	25 (59.5%)	14 (25.5%)	< 0.001***	8 (42.1%)	5 (20.8%)	0.136
Chemotherapeutic agents use, n (%)	8 (19.0%)	27 (49.1%)	0.002**	6 (31.6%)	10 (41.7%)	0.502
Initial symptoms, n (%)						
Fever	30 (71.4%)	32 (58.2%)	0.181	13 (68.4%)	12 (50.0%)	0.229
Cough	19 (45.2%)	23 (41.8%)	0.738	9 (47.4%)	12 (50.0%)	0.865
Dyspnea	27 (64.3%)	21 (38.2%)	0.011*	12 (63.2%)	9 (37.5%)	0.099
Initial laboratory findings, median (IQR)						
PaO_2_/FiO_2_ ratio	258 (219, 297)	305 (275, 335)	0.052	227 (183, 271)	305 (260, 351)	0.014*
WBC, 10^9/L	7.08 (5.36, 9.43)	6.62 (3.94, 10.51)	0.583	6.41 (4.76, 8.75)	5.19 (4.35, 9.74)	0.604
CRP, mg/L	2.16 (0.53, 7.65)	1.19 (0.20, 5.30)	0.033*	2.64 (0.78, 6.10)	1.33 (0.15, 2.64)	0.117
serum LDH, IU/L	373 (302, 557)	257 (181, 357)	< 0.001***	354 (255, 468)	285 (188, 345)	0.060
serum β-D-glucan, pg/mL	284.7 (122.2, 523.3)	19.4 (10.0, 40.7)	< 0.001***	200.4 (100.7, 469.7)	46.6 (11.4, 131.0)	0.005**
Semantic CT features, n (%)						
Ground glass opacity	42 (100.0%)	47 (85.5%)	0.010*	19 (100.0%)	21 (87.5%)	0.114
Interlobular septal thickening	22 (52.4%)	21 (38.2%)	0.165	6 (31.6%)	9 (37.5%)	0.689
Cyst	12 (28.6%)	10 (18.2%)	0.228	7 (36.8%)	3 (12.5%)	0.064
Consolidation	16 (38.1%)	26 (47.3%)	0.369	9 (47.4%)	16 (66.7%)	0.208
Honeycombing	5 (11.9%)	4 (7.3%)	0.438	4 (21.1%)	3 (12.5%)	0.456
Lymphadenopathy	10 (23.8%)	10 (18.2%)	0.499	1 (5.3%)	7 (29.2%)	0.048*
Pleural effusion	7 (16.7%)	15 (27.3%)	0.219	1 (5.3%)	7 (29.2%)	0.048*

*Abbreviations:* *IQR* Interquartile range, *PaO*_*2*_ Partial pressure of oxygen, *FiO*_*2*_ Fraction of Inspired oxygen, *WBC* White blood cell, *CRP *C-reaction protein, *LDH *Lactate dehydrogenase

*P<0.05; **P<0.01; ***P<0.001

### Performance of the radiomics model

Univariate analysis showed that 648 of the 1316 radiomics features differed significantly between the PCP and non-PCP groups of the training cohort. LASSO regression was then performed, and the model had the lowest error when λ = 0.069 and log λ = -1.161 (Figure S1 in Additional file 1), and nine non-zero features were identified to construct the radiomics model (Table S1 in Additional file 1). The radiomics model constructed by the logistic regression was found to perform best in both the training (AUC = 0.950, 95% CI: 0.908–0.992) and validation cohorts (AUC = 0.954, 95% CI: 0.898-1.000) (Figure S2 in Additional file 1). The Radscore of the PCP group were significantly higher than those of the non-PCP group in both the training (2.5 ± 2.4 vs. -3.1 ± 2.7, *P* < 0.001) and validation (2.0 ± 2.8 vs. -4.3 ± 3.0, *P* < 0.001) cohorts. The Radscore was calculated as follows:

$$\text{R}\text{a}\text{d}\text{s}\text{c}\text{o}\text{r}\text{e}=74.172-60.024\times {X}_{1}-10.643\times {X}_{2}+6.850\times {X}_{3}+1.613\times {X}_{4}+1.378\times {X}_{5}-2.394\times {X}_{6}-10.677\times {X}_{7}-2.241\times {X}_{8}+1.276\times {X}_{9}$$

**Fig. 3:**
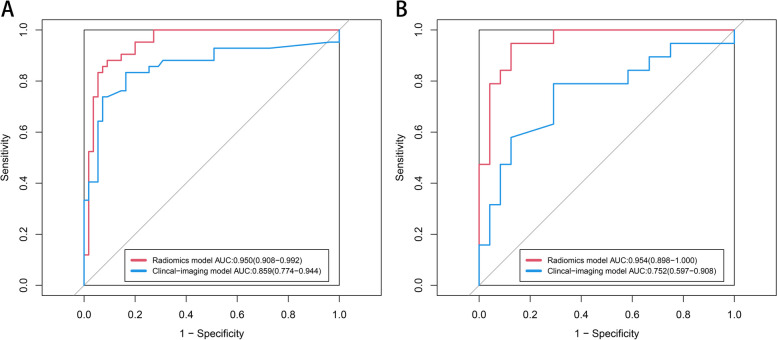
The receiver operating characteristic (ROC) curves of the radiomics model and traditional clinical-imaging model in (**A**) training and (**B**) validation cohorts. The radiomics model exhibited better performance than the traditional clinical-imaging model in both training (*P* = 0.049) and validation cohort (*P* = 0.011). The 95% confidence interval of AUC was shown as the data in the parentheses

**Fig. 4:**
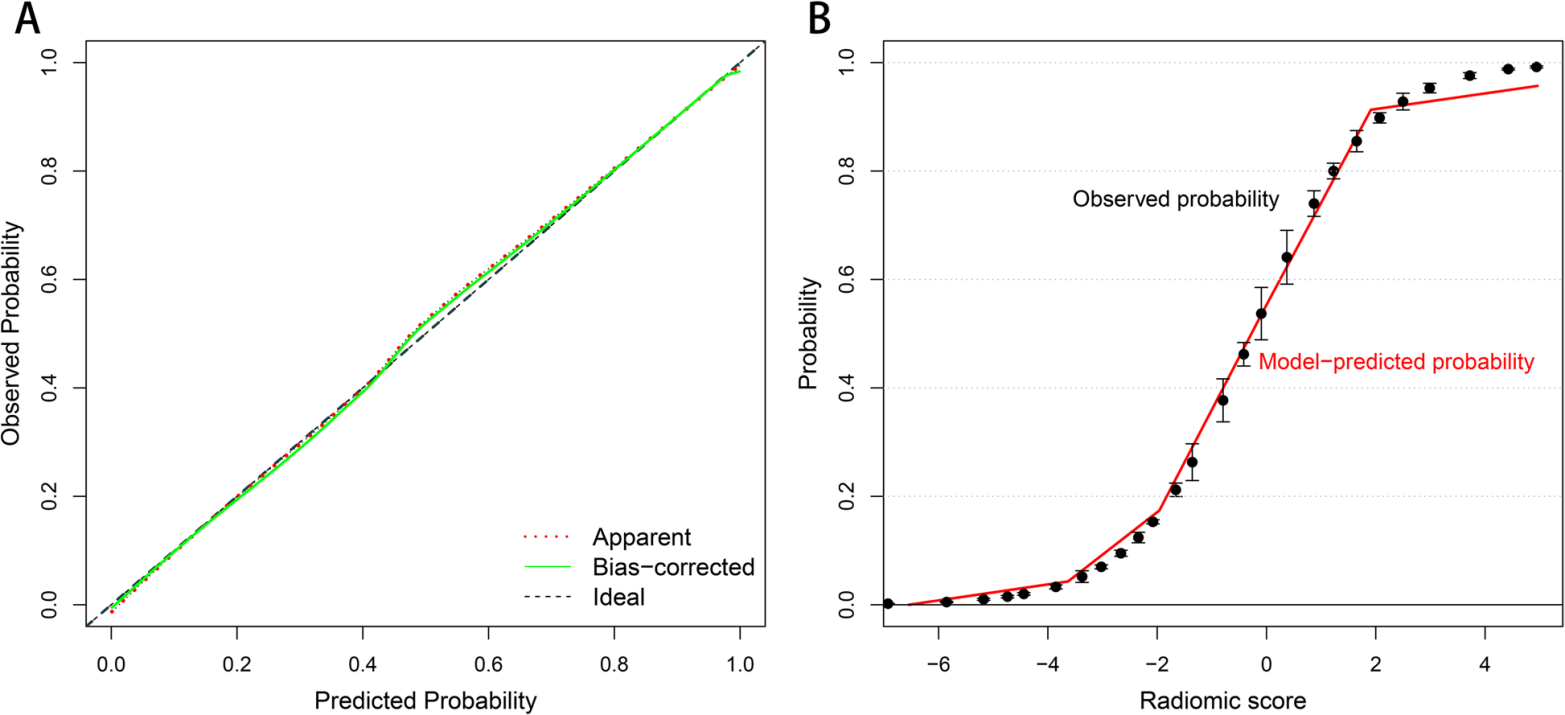
The calibration curves of the radiomics model. **A** The curve showed a good agreement between prediction and observation by 1000 groups bootstrap-resampling. **B** Logistic regression estimated observed probability with 95% confidence interval vs. radiomics model-predicted probability (red line) based on the calculated radiomic score for the diagnosis of *Pneumocystis jirovecii* pneumonia

The radiomics model showed a more efficient diagnosis performance than the traditional model with a higher AUC in the training (0.950 vs. 0.859, *P* = 0.049) and validation cohorts (0.954 vs. 0.752, *P* = 0.011) (Fig. 3). The calibration curves showed good consistency between predictions and observations (corrected C-index: 0.948) (Fig. 4). The waterfall plot of the Radscore also showed that radiomics model could distinguish most PCP patients from non-PCP ones (Figure S3 in Additional file 1).

### Clinical utility of the radiomics model

**Fig. 5:**
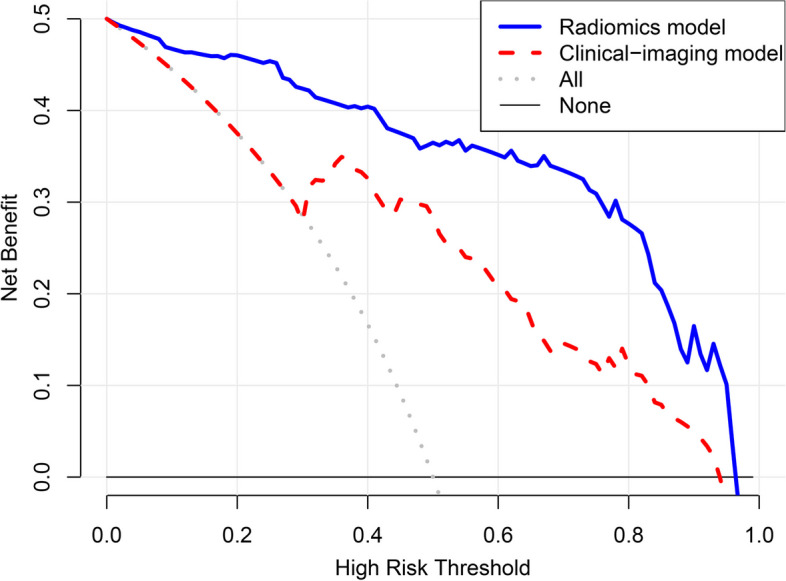
Decision curve analysis of the radiomic model and the traditional clinical-imaging model. The curve showed that using the radiomics model added more net benefit than the traditional model in differentiating *Pneumocystis jirovecii* pneumonia from other types of pneumonia over a threshold probability range of 0–95%

The decision curve analysis indicated that the use of radiomics model added more net benefit than traditional model in differentiating PCP from non-PCP over a threshold probability range of 0–95% (Fig. 5).

### Performance comparison with serum β-D-glucan and LDH

In all patients, the diagnostic performance of the radiomics model and serum β-D-glucan/LDH previously used for diagnosis were calculated (Table 3), the radiomics model had the highest diagnostic accuracy (90.0%).

**Table 3:** Diagnostic performance of the radiomics model compared with -D-glucan and LDH for the diagnosis of *Pneumocystis jirovecii* pneumonia

**Variables**	**Accuracy**	**Sensitivity**	**Specificity**	**Positive predictive value**	**Negative predictive value**
Radiomics model	90.0%	90.2%	89.9%	87.3%	92.2%
β-D-glucan > 200 pg/ml	77.9%	60.7%	91.1%	84.1%	75.0%
β-D-glucan < 80 pg/ml	79.3%	80.3%	78.5%	74.2%	83.8%
LDH > 300 IU/ml	65.7%	72.1%	60.8%	58.7%	73.8%

*Abbreviation:* *LDH *Lactate dehydrogenase

### Combining strategy for PCP diagnosis

**Fig. 6:**
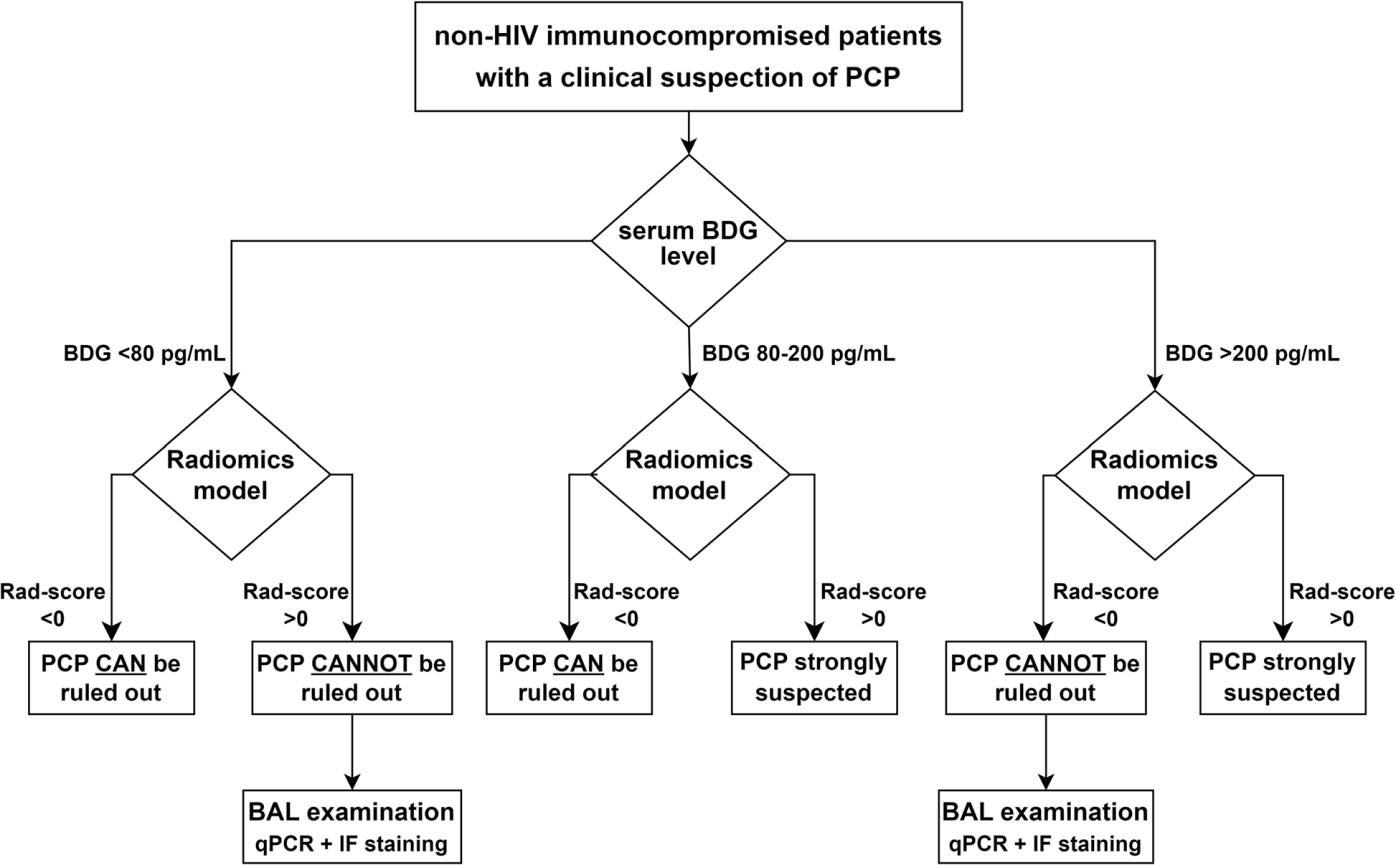
A diagnostic strategy combining radiomics model and serum BDG for the diagnosis of *Pneumocystis jirovecii* pneumonia. HIV, human immunodeficiency virus; PCP, *Pneumocystis jirovecii* pneumonia; BDG, β-D-glucan; BAL, bronchoalveolar lavage; qPCR, quantitative polymerase chain reaction; IF: immunofluorescent

Table 4. showed the diagnostic performance of the radiomics model in patients with different serum β-D-glucan levels. In patients with serum β-D-glucan > 200 pg/mL, the PPV of the radiomics model was 91.9% and in patients with serum β-D-glucan < 80 pg/mL, the NPV was 96.6%. In addition, among patients with serum β-D-glucan of 80–200 pg/mL, the PPV and NPV of the radiomics model were 100.0% and 90.9%, respectively. Therefore, we developed a new diagnostic strategy for PCP by combining radiomics model and serum β-D-glucan levels (Fig. 6). In our study, 118 of 140 patients (84.3%) could be diagnosed by this method, with a diagnostic accuracy of 95.8% for PCP (sensitivity: 93.8%, specialty: 97.1%, PPV: 95.7%, NPV: 95.8%).

**Table 4:** Diagnostic performance of the radiomics model in different serum -D-glucan levels for the diagnosis of *Pneumocystis jirovecii* pneumonia

**Variables**	**Accuracy**	**Sensitivity**	**Specificity**	**Positive predictive value**	**Negative predictive value**
Radiomics model					
In β-D-glucan > 200 pg/ml	78.6%	55.7%	96.2%	91.9%	73.8%
In β-D-glucan < 80 pg/ml	82.9%	96.7%	72.2%	72.8%	96.6%
In β-D-glucan of 80–200 pg/ml	95.5%	91.7%	100.0%	100.0%	90.9%

*Abbreviation:* *BDG *β-D-glucan

## Discussion

In this study, we constructed a CT-based radiomics model to identify the risk of PCP infection in non-HIV patients with CT manifestations of pneumonia. The established radiomics model demonstrated better diagnostic efficiency than traditional risk factors in clinical characteristics and CT findings. We also developed a diagnostic strategy based on the radiomics model and serum β-D-glucan levels, which may identify the risk of PCP infection in non-HIV patients accurately and non-invasively.

The CT manifestations of PCP are commonly considered to be non-specific [5, 14]. Nevertheless, some investigators believed that CT features might be of some value in diagnostic decision-making. A nested case-control study involving 72 PCP patients and 288 non-PCP patients showed that ‘increased interstitial markings’ and ‘ground glass opacity’ were independently associated with the diagnosis of PCP, whereas ‘pleural effusion’ and ‘nodular findings’ were independently negatively associated [14]. They developed a nomogram to predict the post-CT probability of PCP and to assist clinical practice (i.e., non-invasive testing in low risk patients and more invasive testing in high risk patients). In our study, the PCP group had more ground glass opacity, more cysts and fewer pleural effusion, but none of these were independent risk factors for diagnosis by multivariate logistic regression analysis.

In this study, serum β-D-glucan was the only independent traditional risk factor for the diagnosis of PCP. Morjaria et al. suggested that β-D-glucan > 200 pg/mL had 100% sensitivity and 100% PPV for PCP diagnosis in cancer patients [24]. A meta-analysis by Del Corpo et al. showed a pooled sensitivity of 86% for β-D-glucan in the non-HIV patients, and an NPV of 95% for β-D-glucan at < 80 pg/mL even at a prevalence rate of 50% [12]. The diagnostic accuracy of β-D-glucan seemed to be not enough for diagnosis. In our study, the serum β-D-glucan also exhibited unsatisfied stability and moderate performance in the validation cohort (AUC = 0.752). Therefore, clinical characteristics and CT semantic features may be not enough for PCP diagnosis due to insufficient diagnostic efficacy.

Radiomic analysis has been wildly used in cancer research [15, 26–28] and in the diagnosis of COVID-19 pneumonia [29–31]. For the diagnosis of PCP, we identified only one study conducted by Kloth et al. that was relevant to the radiomic features of PCP [19]. They explored CT-textures of one or two local regions with typical disease manifestations in 21 patients with PCP (including non-HIV patients). Eleven first- and second-order texture features were analyzed but no specific features were found for diagnostic purposes. Differently, the radiomics model constructed in our study performed well. There are three possible reasons for the difference in results between Kloth’s study and ours. First, the different sample sizes of the studies (21 versus 140) affected the accuracy of the diagnoses. Second, the ROIs in Kloth’s study were obtained from localized squares drawn by a senior reader in the “diseased area”. In our study, we obtained the whole pneumonia regions in the lungs. Third, we analyzed more radiomics features, including wavelet features and LOG features, which were interestingly all the features selected and incorporated into our radiomic model. These features were obtained by filtering the original image to enhance some special features such as edge regions [32]. The decision curve analysis showed a good clinical value of the radiomics model, meaning that radiomics could be used as a tool to assist clinicians in the diagnosis of PCP.

In addition, we calculated the diagnostic performance of serum β-D-glucan and LDH mentioned in previous studies for the diagnosis of PCP [1, 12, 24]. Compared to these previously used clinical indicators, the radiomics model performed best. Furthermore, we assessed the diagnostic efficacy of radiomics at different β-D-glucan levels. Based on the results, we established a strategy for non-invasive diagnosis of PCP, which could identify the risk of PCP infection in non-HIV patients with a diagnostic accuracy of 95.8%.

Nowadays, qPCR and IF staining tests of BAL fluid samples are the primary tests recommended by the guidelines to confirm the diagnosis of PCP in non-HIV patients, with serum β-D-glucan testing as an adjunctive laboratory diagnostic tool [8]. Although well-tolerated, it is not uncommon for patients to develop fever and worsening hypoxemia after the bronchoscopy [33]. The implementation of BAL techniques also requires specialist technicians, specialized equipment and rooms, and is difficult to carry out in resource-limited areas and medical centers. Other non-invasive methods, such as qPCR and IF staining of upper respiratory specimens (i.e., induced sputum [34, 35], oral washings [36, 37], nasopharyngeal aspirate [38, 39]) and/or blood samples [38, 40] are not diagnostically satisfactory, with the diagnostic specificity ranged from 54 to 100%, and the diagnostic sensitivity from 50 to 77%. The new strategy in our study, if its general validity is confirmed in future studies, has the potential to provide an accurate and non-invasive way to identify the risk of PCP infection in non-HIV patients.

This study had several limitations. First, the sample size of the population included in this study was not large. Second, this single-center retrospective study did not include external validation, which may have led to bias in model performance. Third, six patients with qPCR (-) and IF (+) were excluded to ensure confirmation of pathological results (as the results were technically inconsistent), which could have led to a small selection bias. Fourth, PPV and NPV were calculated in the existing population (PCP prevalence of 43.6%) and may be altered due to changes in PCP prevalence. Therefore, a large multicenter study is needed to validate these findings.

## Conclusions

Radiomics showed good diagnostic performance in differentiating PCP from other types of pneumonia in non-HIV patients. A combined diagnostic method including radiomics and serum β-D-glucan has the potential to provide an accurate and non-invasive way to identify the risk of PCP infection in non-HIV patients with CT manifestation of pneumonia.

### Supplementary Information

**Additional file 1: Table S1.** Selected radiomics features by logistic regression in the training cohort. **Figure S1.** Radiomic features screening using the Least Absolute Shrinkage and Selection Operator (LASSO). (A) Tenfold cross-validation analysis showed that the model error was minimized when λ = 0.069 and log λ = -1.161 (the first vertical dashed line), and nine non-zero features were screened out. (B) The coefficient profiles of the 1316 features. **Figure S2.** Receiver operating characteristic (ROC) curves of radiomics models constructed by logistic regression (LR), support vector machine (SVM), adaboost (AB) and decision tree (DT) in the training (A) and validation (B) cohorts. LR exhibited the best performance (area under the curve (AUC) = 0.954) in the validation cohort. The 95% confidence interval of AUC was shown as the data in the parentheses. **Figure S3.** Waterfall plot of the Radscore for the radiomics model. The horizontal axis represented all patients (*n *= 140) and the vertical axis represented the Radscore calculated by logistic regression. patients with *Pneumocystis jirovecii* pneumonia (PCP) were marked in red and patients with other types of pneumonia (non-PCP) were marked in blue. It can be seen that most PCP patients had higher scores and most non-PCP patients had lower scores. PCP, *Pneumocystis jirovecii* pneumonia.

## Data Availability

The datasets used and analyzed during the current study are available from the corresponding author on reasonable request.

## Abbreviations

AUC: Area under the curve
BAL: Bronchoalveolar lavage
CT: Computed tomography
HIV: Non-human immunodeficiency virus
IF: Immunofluorescent
LASSO: Least Absolute Shrinkage and Selection Operator
LDH: Lactate dehydrogenase
LoG: Laplacian of Gaussian
NPV: Negative predictive value
PCP: *Pneumocystis jirovecii* pneumonia
PPV: Positive predictive value
qPCR: Polymerase chain reaction
Radscore: Radiomic score
ROC: Receiver operating characteristic curve
