# Supplementary material for: Computed tomography-based radiomics improves non-invasive diagnosis of Pneumocystis jirovecii pneumonia in non-HIV patients: a retrospective study
Source: BMC Pulm Med. 2024 Jan 2;24:11. doi: 10.1186/s12890-023-02827-4 (PMC10762815; doi:10.1186/s12890-023-02827-4)
Supplement: Supplementary file 1 — Additional file 1: Table S1. Selected radiomics features by logistic regression in the training cohort. Figure S1. Radiomic features screening using the Least Absolute Shrinkage and Selection Operator (LASSO). (A) Tenfold cross-validation analysis showed that the model error was minimized when λ = 0.069 and log λ = -1.161 (the first vertical dashed line), and nine non-zero features were screened out. (B) The coefficient profiles of the 1316 features. Figure S2. Receiver operating characteristic (ROC) curves of radiomics models constructed by logistic regression (LR), support vector machine (SVM), adaboost (AB) and decision tree (DT) in the training (A) and validation (B) cohorts. LR exhibited the best performance (area under the curve (AUC) = 0.954) in the validation cohort. The 95% confidence interval of AUC was shown as the data in the parentheses. Figure S3. Waterfall plot of the Radscore for the radiomics model. The horizontal axis represented all patients (n = 140) and the vertical axis represented the Radscore calculated by logistic regression. patients with Pneumocystis jirovecii pneumonia (PCP) were marked in red and patients with other types of pneumonia (non-PCP) were marked in blue. It can be seen that most PCP patients had higher scores and most non-PCP patients had lower scores. PCP, Pneumocystis jirovecii pneumonia. [file 12890_2023_2827_MOESM1_ESM.docx]

**SUPPLEMENTARY MATERIALS**

**Tables**

| **Table S1.** Selected radiomics features by logistic regression in the training cohort | | |
| --- | --- | --- |
| **Variables** | **Radiomic features** |  |
| X_1_ | log-sigma-3-0-mm-3D_glcm_Contrast |  |
| X_2_ | log-sigma-1-0-mm-3D_glcm_Correlation |  |
| X_3_ | wavelet-LLL_glcm_MCC |  |
| X_4_ | log-sigma-2-0-mm-3D_glrlm_RunLengthNonUniformity |  |
| X_5_ | log-sigma-3-0-mm-3D_firstorder_MeanAbsoluteDeviation |  |
| X_6_ | log-sigma-4-0-mm-3D_firstorder_MeanAbsoluteDeviation |  |
| X_7_ | log-sigma-4-0-mm-3D_glszm_SizeZoneNonUniformityNormalized |  |
| X_8_ | log-sigma-5-0-mm-3D_glszm_SmallAreaEmphasis |  |
| X_9_ | wavelet-LLL_ngtdm_Busyness |  |

**
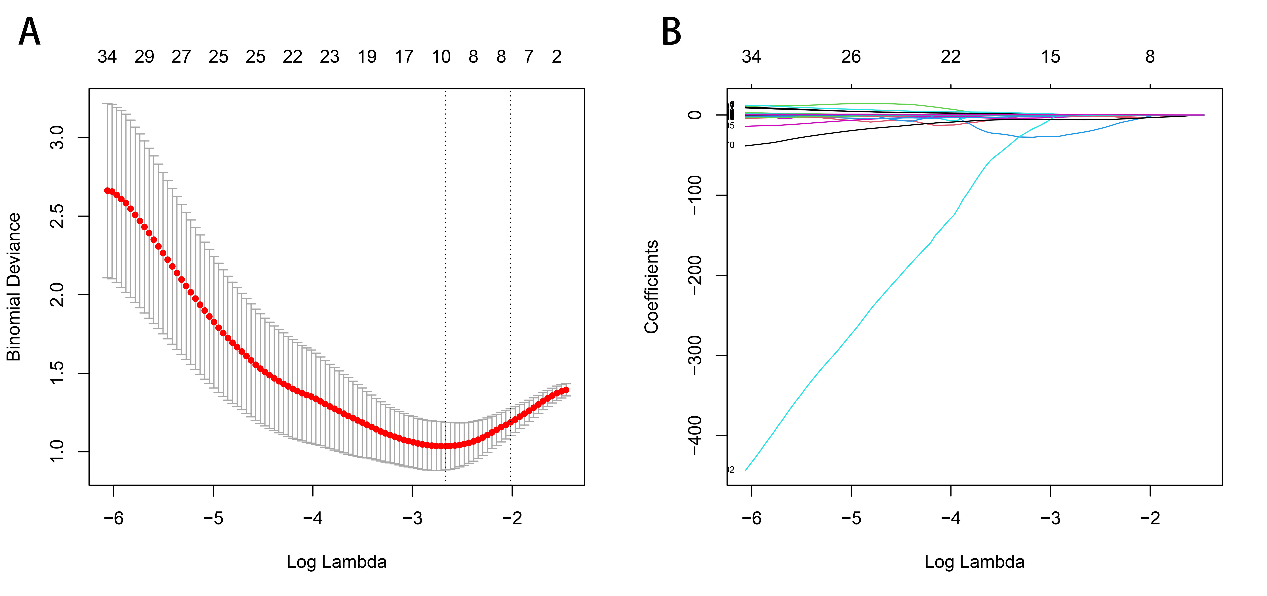
Figures**

**Figure S1.** Radiomic features screening using the Least Absolute Shrinkage and Selection Operator (LASSO). (**A**) Tenfold cross-validation analysis showed that the model error was minimized when λ = 0.069 and log λ = -1.161 (the first vertical dashed line), and nine non-zero features were screened out. (**B**) The coefficient profiles of the 1316 features.

**
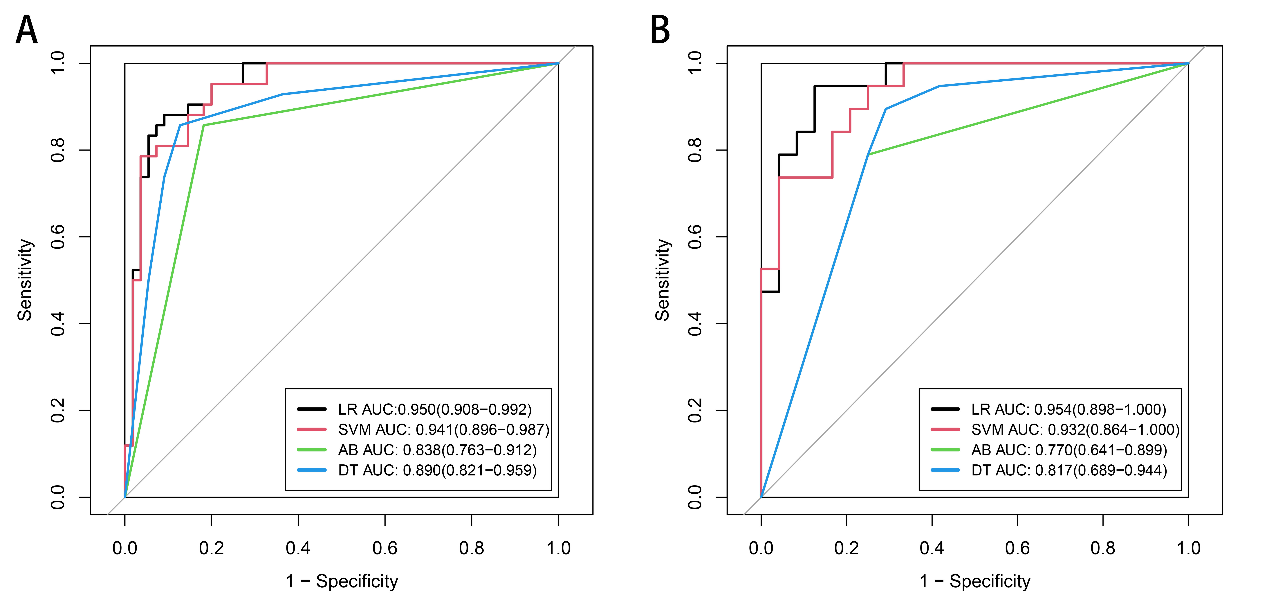
**

**Figure S2.** Receiver operating characteristic (ROC) curves of radiomics models constructed by logistic regression (LR), support vector machine (SVM), adaboost (AB) and decision tree (DT) in the training (**A**) and validation (**B**) cohorts. LR exhibited the best performance (area under the curve (AUC) = 0.954) in the validation cohort. The 95% confidence interval of AUC was shown as the data in the parentheses.

**
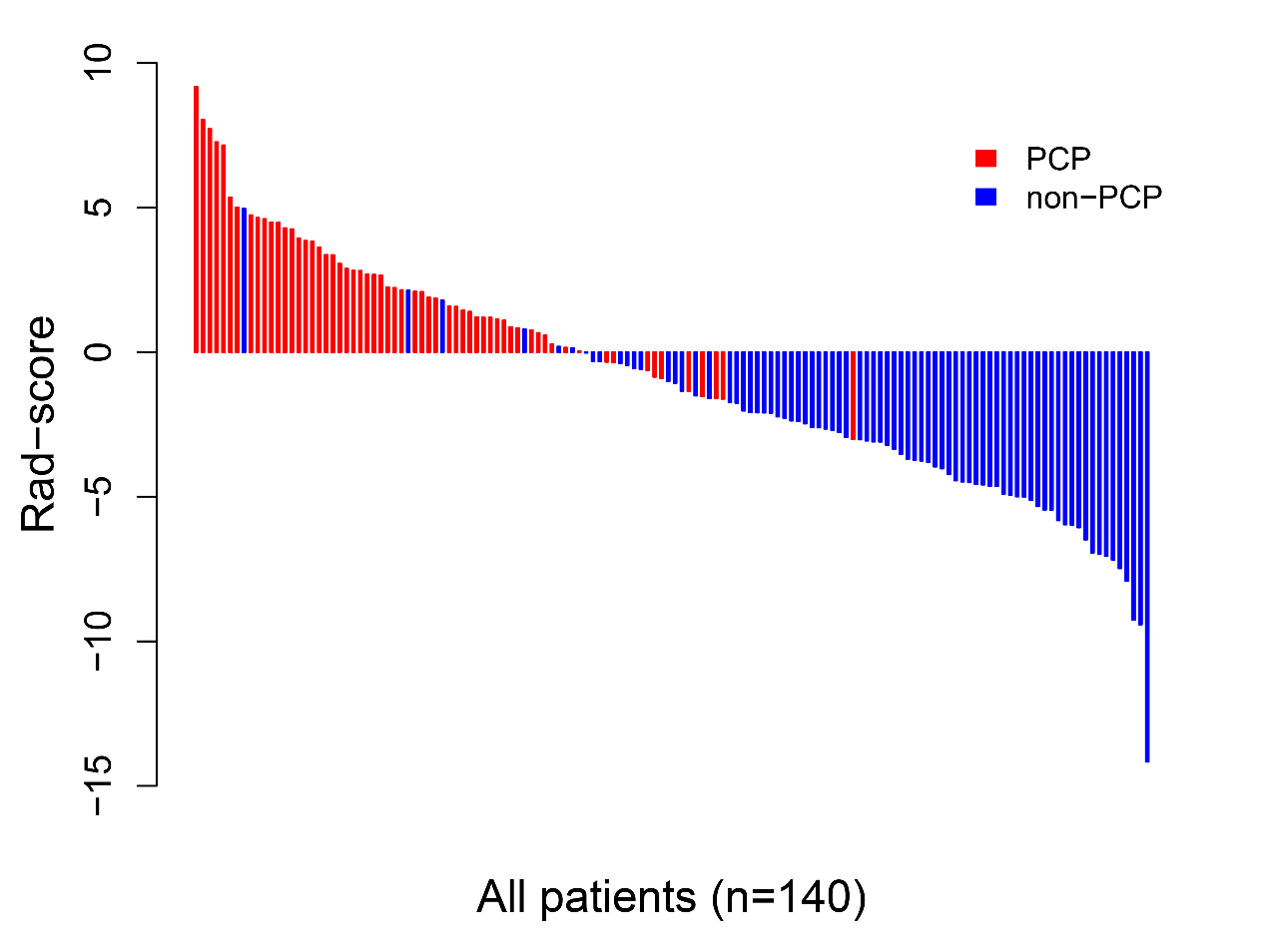
**

**Figure S3.** Waterfall plot of the Radscore for the radiomics model. The horizontal axis represented all patients (n=140) and the vertical axis represented the Radscore calculated by logistic regression. patients with *Pneumocystis jirovecii* pneumonia (PCP) were marked in red and patients with other types of pneumonia (non-PCP) were marked in blue. It can be seen that most PCP patients had higher scores and most non-PCP patients had lower scores. PCP, *Pneumocystis jirovecii* pneumonia.
